# Heat-inactivated *Lactobacillus plantarum* nF1 promotes intestinal health in Loperamide-induced constipation rats

**DOI:** 10.1371/journal.pone.0250354

**Published:** 2021-04-19

**Authors:** Seon-Ah Park, Geum-Hwa Lee, The-Hiep Hoang, Hwa-Young Lee, In-Yeong Kang, Myong-Ja Chung, Jong-Sik Jin, Han-Jung Chae

**Affiliations:** 1 Non-Clinical Evaluation Center, Research Institute of Clinical Medicine of Jeonbuk National University, Biomedical Research Institute of Jeonbuk National University Hospital, Jeonju, South Korea; 2 Department of Pharmacology and Institute of New Drug Development, School of Medicine, Jeonbuk National University, Jeonju, South Korea; 3 Biogenicskorea Co., Ltd., Seocho-gu, Seoul, Korea; 4 Department of Pathology, Jeonbuk National University Medical School, Jeonju, Korea; 5 Department of Oriental Medicine Resources, Jeonbuk National University, Iksan, South Korea; University of Nevada School of Medicine, UNITED STATES

## Abstract

Constipation is a common condition that affects individuals of all ages, and prolonged constipation needs to be prevented to avoid potential complications and reduce the additional stress on individuals with pre-medical conditions. This study aimed to evaluate the effects of heat-inactivated *Lactobacillus plantarum* (HLp-nF1) on loperamide-induced constipation in rats. Constipation-induced male rats were treated orally with low to high doses of HLp-nF1 and an anti-constipation medication Dulcolax for five weeks. Study has 8 groups, control group; loperamide-treated group; Dulcolax-treated group; treatment with 3.2 × 10^10^, 8 × 10^10^ and 1.6 × 10^11^, cells/mL HLp-nF1; Loperamide + Dulcolax treated group. HLp-nF1 treated rats showed improvements in fecal pellet number, weight, water content, intestinal transit length, and contractility compared to the constipation-induced rats. Also, an increase in the intestine mucosal layer thickness and the number of mucin-producing crypt epithelial cells were observed in HLp-nF1-treated groups. Further, the levels of inflammatory cytokines levels were significantly downregulated by treatment with HLp-nF1 and Dulcolax. Notably, the metagenomics sequencing analysis demonstrated a similar genus pattern to the pre-preparation group and control with HLp-nF1 treatment. In conclusion, the administration of >3.2 × 10^10^ cells/mL HLp-nF1 has a positive impact on the constipated rats overall health.

## Introduction

People’s lifestyles are rapidly changing, and changing lifestyles have brought changes in dietary patterns, which have led to a higher prevalence of constipation. Several reports suggest that individuals having colon or rectum problems, hormonal changes, and diabetes potentially contribute to the risk of constipation. Prolonged constipation needs to be prevented to avoid potential complications and reduce the additional stress on individuals with pre-medical conditions. Advancement of science has shed light on the relation between constipation and gut microbiota, suggesting that it may disturb crosstalk between the intestinal microbiota, the immune system, and intestinal permeability [1, 2]. Until recently, constipation treatment focused on intestinal function; however, recent developments have suggested gut microbiota involvement, which opens opportunities to treat constipation through improved balance in gut microbiota. In line with these developments, several studies have revealed that a decrease in beneficial bacteria such as *Bifidobacterium* or *Lactobacillus* and an increase in pathogens [3] might contribute to constipation directly or indirectly. Hence, supplementation with these beneficial species like *Bifidobacteria* or *lactobacilli* as a probiotic could prevent or treat constipation. Also, probiotics not only treat constipation but also offers several other health benefits beyond nutritive functions.

Probiotics are generally safe since the correlation between probiotic use and adverse events is low regarding their widespread use [4, 5]. Thus, probiotics are the most popular option to promote a healthy balance of gut microbiota. It has been a therapeutic option for various clinical symptoms such as diarrhea, constipation associated with irritable bowel syndrome (IBS), colitis, and allergies. However, laxatives and stool softeners are commonly used as over-the-counter remedies for constipation. Nevertheless, efforts have been continued to standardize the use of probiotics in constipation management. There are few commercially available probiotic supplements such as Physician’s Choice, Phillip’s Daily Care, and Earth’s pearl to treat constipation even though clinician’s prescription of probiotics is inconsistent, nonspecific, and sometimes only upon patient request [6]. However, probiotics are not completely safe due to the possibility of viable bacteria as a source of infection [4, 5] and individual susceptibility against live probiotics with unknown etiology. There are few reports about the translocation of bacteria from the gut to the systemic circulation, which may cause infection and lead to clinical complications. These drawbacks of probiotic use have led to exploring the use of non-viable heat-killed probiotics [7–9].

Consequently, several reports have shown that preparations containing inactivated or dead cells can maintain similar biological responses, as seen with live cells [9, 10]. Several animal investigations revealed comparable effects of viable and inactivated probiotics on innate immunity [11]. Furthermore, clinical studies have suggested that non-viable probiotic fractions can modulate the human immune system [12]. In another clinical study on the consumption of heat-inactivated *Lactobacillus* cells in a dairy drink, bowel movement frequencies improved scores for several evacuations and fecal odors [13]. These results indicate the potential efficacy of non-viable *Lactobacillus* cells against constipation. Heat-inactivated *Lactobacillus gasseri* also had a favorable effect on physical symptoms such as sleep quality in a clinical study [14], potentially related to constipation. Nevertheless, the bacterial viability state is required for the probiotic effect to benefit, especially intestinal microbiota. Therefore, the objective of the present study is to investigate the effects of heat-inactivated *L*. *plantarum* (HLp-nF1) on loperamide (Lop)-induced constipation in rats by examining changes in their intestinal microbiota, inflammatory substances, and intestinal contractility.

## Materials and methods

### Animal feed and HLp-nF1oral administration

The current study was carried out by strictly following the Principles of Laboratory Animal Care of Association for Assessment and Accreditation of Laboratory Animal Care International (AAALAC) of the Jeonbuk National University Hospital. The protocols used in this study were approved by the Institutional Animal Care and Use Committee (IACUC) of the Jeonbuk National University Hospital (Ethical approval number cuh-IACUC-2018-1-1). Male Sprague–Dawley rats (~260g) were purchased from Saeronbio Inc. (Uiwang-si, Gyeonggi-do, Korea). The rats were housed at 22±2°C under a 12 h light/dark cycle, and they were allowed access to food and water ad libitum. All the rats received a commercial standard chow diet having crude protein 15.2%, crude fiber 4%, crude fat 3.2%, Calcium 0.68%, and sodium 0.22% (Biogenics, Korea) during the study.

After environmental acclimation for one week, rats were randomly separated into 8 groups, consisting of 12 rats in each group. The rats were orally administered Hlp-nF1 at different doses (Low, 3.2 × 10^10^; Medium, 8×10^10^; High, 1.6 × 10^11^ cells/mL) for five weeks. The vehicle for the HLp-nF1 is ultra-pure water (Invitrogen). The rats were treated with the HLp-nF1 ml/rat using 3 mL Syringe with a disposable feeding needle (Fuchigami Kikai, Japan). The loperamide (Lop, 2.5 mg/kg; Sigma, MO, USA) group rats were intraperitoneally (IP) injected with a single dose of freshly prepared Lop for one week before sacrifice to obtain constipation rat model. Additionally, the other rats were orally injected with Dulcolax (0.75 mg/kg; Sigma-Aldrich) or Lop alone for five weeks. Together, study has 8 groups Con, control group; Lop, loperamide-treated group; Dul, Dulcolax-treated group; HHL, treatment with 1.6 × 10^11^ cells/mL HLp-nF1; Lop+LHL, treatment with loperamide and 3.2 × 10^10^ cells/mL HLp-nF1; Lop+MHL, treatment with loperamide and 8 × 10^10^ cells/mL HLp-nF1; Lop+HHL, treatment with loperamide and 1.6 × 10^11^ cells/mL HLp-nF1; Lop+Dul treated group. S1A represents the experimental scheme followed in the study.

### Sample preparation

HLp-nF1was obtained from Biogenics Korea Co., Ltd. (Seoul, Korea). It is manufactured by incubating *L*. *plantarum* for 20h under controlled pH, followed by incubation at high temperature, high salinity (1.0%, w/w), and low pH (pH 5.0) for 4h. It is then sterilized at 80°C for 10 min and nano-dispersed by high-pressure homogenization.

### Food, water intake, body weight, and fecal excretion

Food and water intake, fecal parameters, and body weight were measured once a week before Lop injection, and then the fecal parameters were measured every day during the study period. Fecal pellets were collected using filter paper bedding in the cages for 24h. The fecal pellets water content was calculated as the difference between the wet and dry weights of pellets collected over a period of 24h.

### Biochemical analysis

Blood glucose level was measured using blood samples collected from the rat tail before the oral administration of HLp-nF1and sacrifice. Before sacrifice, about 10 mL of blood samples were collected from the abdominal artery after anesthetizing the rats with ketamine. Approximately 10 mL of blood was collected from each rat. The collected blood was transferred to serum-separating or plasma-separating tubes (BD Bioscience, Franklin Lakes, NJ, USA) and placed on a rotator (Vision, Scientific Co., Ltd., Daejeon, Korea) for 5 min at room temperature (RT, 20°C). The blood samples were centrifuged at 2000 × g for 20 min at RT for separating serum or plasma. The level of total cholesterol, triglyceride, tumor necrosis factor (TNF)α, interferon (IFN)γ, interleukin (IL)-1β, IL-6, IL-10, IL-12, cyclooxygenase (COX)-2, prostaglandin E2 (PGE2), and calprotectin was measured using ELISA kits (Thermo Fisher, Waltham, MA, USA and MyBiosource, San Diego, CA, USA).

### Measurement of gastrointestinal motility and tissue sampling

Measurement of intestinal parameters and sampling of tissues were performed as described previously [15]. Briefly, Barium sulfate (Daejung Chemicals & Metals Co. LTD., Siheung-si, Gyeonggi-do, Korea) was orally administered to all the rats after 12 h of fasting. Later, the intestinal transit length was measured, and specimens were collected for further analysis. Intestinal transit was calculated as follows,

$$\text{Intestinal}\;\text{transit}(\%)=\frac{\text{A}(\text{pyloric}∼\text{barium}\;\text{sulfate}\;\text{terminal})}{\text{B}(\text{pyloric}∼\text{ileum}\;\text{terminal})}\times 100$$

### Western blotting

Immunoblotting was performed as described previously [16]. Briefly, dissected intestinal were homogenized and lysed with lysis buffer. Protein extract was separated on SDS-PAGE and transferred to polyvinylidenedifluoride (PVDF) membrane and probed with the following antibodies: IL-6, IL-1β, TNF-α, PGE2, COX-2, and β-actin from Santa Cruz Biotechnology (Santa Cruz, CA). Band intensity was determined by densitometry analysis using the ImageJ analysis system.

### Hematoxylin and Eosin (H&E) and Alcian blue staining

Hematoxylin and eosin (H&E) staining was performed as described previously [16]. Briefly, processed paraffin-embedded tissues were sectioned, deparaffinized, rehydrated, and washed with water. Later, stained with H&E and dehydrated and observed under a microscope. The sections were also stained with Alcian blue at pH 2.5, as described previously [17]. The intestine muscle thickness and crypt area changes were determined by calculating the average volume of three regions per section selected using a light microscope. Stained sections were observed for hematoxylin and eosin (H&E) and Alcian blue-positive mucus layer using Leica DM 750 (Leica, Wetzlar, Germany) and analyzed using Image J analysis (Oxford instruments, Abingdon-on-Thames, UK).

### Immunohistochemistry

Immunohistochemistry was performed as described previously [16]. Formalin-fixed, paraffin-embedded tissue sections were deparaffinized and rehydrated in xylene, followed by decreasing ethanol concentration. Antigen retrieval was accomplished using Target Retrieval Solution in a decloaking chamber (Biocare Medical, Concord, CA, USA). To analyze the expression of IL-6, TNF-α, PGE2, COX-2, IL-10, and IL-12, tissue was incubated with the corresponding antibodies (1:100, Santa Cruz Biotechnology, USA). Expressions were detected using 3-amino-9-ethylcarbazole (AEC) substrate chromogen before counterstaining with Harris hematoxylin.

### Measurement of excised ileum segments

Measurements of ileum segments were done as described previously [18]. The ileum segments (15 mm in length) were suspended in a four-channel organ bath containing 4 mL of oxygenated (95% O_2_ and 5% CO_2_) normal physiological salt solution and maintained at 37°C. Each segment’s proximal ends were connected to a displacement transducer (AD Instruments, Colorado Springs, CO, USA) to record contractions from the longitudinal axis. The mechanical activity was digitized on an A/D converter and then visualized, recorded, and analyzed using the Power-Lab/400 System (AD Instruments, Colorado Springs, CO, USA) [15]. The contraction or relaxation of segments was calculated by changes in tension recovery by Ach or PE application.

### Detection of calprotectin in feces

According to the manufacturer’s instructions, the collected feces were left at RT for 30 min, homogenized using Dulbecco’s phosphate-buffered saline (DPBS), and centrifuged at 12000 × g for 10 min to obtain the supernatant. Calprotectin in the feces samples was extracted using commercial enzymatic assay kits (MyBioSource, San Diego, CA, USA).

### Extracellular vesicle isolation and DNA extraction from rat feces samples

Rat feces samples were sent to MD Healthcare, Republic of Korea for DNA metagenomics sequencing. The rat feces sample was filtered through a cell strainer after diluting in 10 mL of PBS for 24 h. Extracellular vesicles (EVs) from the feces samples were isolated by differential centrifugation at 10,000 ×g for 10 min at 4°C. After centrifugation, the pellet (mainly bacteria) and supernatant (mainly EV) were obtained. Bacteria and foreign particles were eliminated by passing the supernatant through a 0.22-μm filter. To extract DNA from the bacteria and EV membrane, bacteria and EVs were boiled for 40 min at 100°C. To eliminate remaining floating particles and waste, the sample was centrifuged at 16,200 × g for 30 min at 4°C, to collect the supernatant. DNA was extracted using a DNA isolation kit (PowerSoil DNA Isolation Kit; MO BIO, Carlsbad, CA, USA), according to the instruction in the manual. DNA from bacteria and EVs in each sample was quantified using a QIAxpert system (QIAGEN, Hilden, Düsseldorf, Germany).

### Bacterial metagenomic sequencing using DNA from rat feces samples

Bacterial genomic DNA was amplified using the primers 16S_V3_F (5’-TCGTCGGCAGCGTCAGATGTGTATAAGAGACAGCCTACGGGNGGCWGCAG-3’) and (5’-GTCTCGTGGCTCGGAGATGTGTATAAGAGACAGGACTACHVGGGTATCTAATCC-3’), which are specific for the V3-V4 hypervariable regions of the 16S rDNA gene [19, 20]. Libraries were prepared using the polymerase chain reaction products according to the MiSeq System guide (Illumina Inc., San Diego. CA, USA) and quantified using QIAxpert (QIAGEN). Each amplicon was quantified; the equimolar ratio was set, pooled, and sequenced on MiSeq (Illumina), according to the manufacturer’s recommendations.

### Analysis of bacterial composition in the microbiota

For bacterial composition, all the samples in a group were mixed to make one sample per group. Prepared samples were sent for analysis. Before the start of the experiment, the feces samples were collected and stored at -70°C. Feces samples were collected from each group on day 6 after Lop administration and were analyzed. Raw pyrosequencing reads were obtained and filtered according to the barcode and primer sequences using MiSeq (Illumina). A taxonomic assignment was performed using the profiling program MDx-Pro ver.1 (MD Healthcare, Korea). Later, high-quality sequencing reads were selected after checking the read length (≥ 300 bp) and quality score (average Phred score ≥ 20). Operational taxonomy units were clustered using the sequence clustering algorithm CD-HIT. Subsequently, the taxonomy assignment was carried out using UCLUST and QIIME against the 16S rDNA sequence database in GreenGenes 8.15.13. Based on sequence similarities, all 16S rDNA sequences were assigned to the following taxonomic levels. The bacterial composition at each level was plotted in the stack bar. In case clusters could not be assigned at the genus level due to a lack of sequences or redundant sequences in the database, the taxon was assigned to the higher level, which is indicated in parentheses.

### Statistical analysis

The results are presented as mean ± SD. Significance between the means of two groups were determined using a t-test. Statistical calculations, plotting, and curve fittings were performed using Prism 5 (GraphPad Software, San Diego, CA, USA). The results with P-value < 0.05 were considered significant.

## Results

### Effect of HLp-nF1 on constipation markers

**Fig 1:**
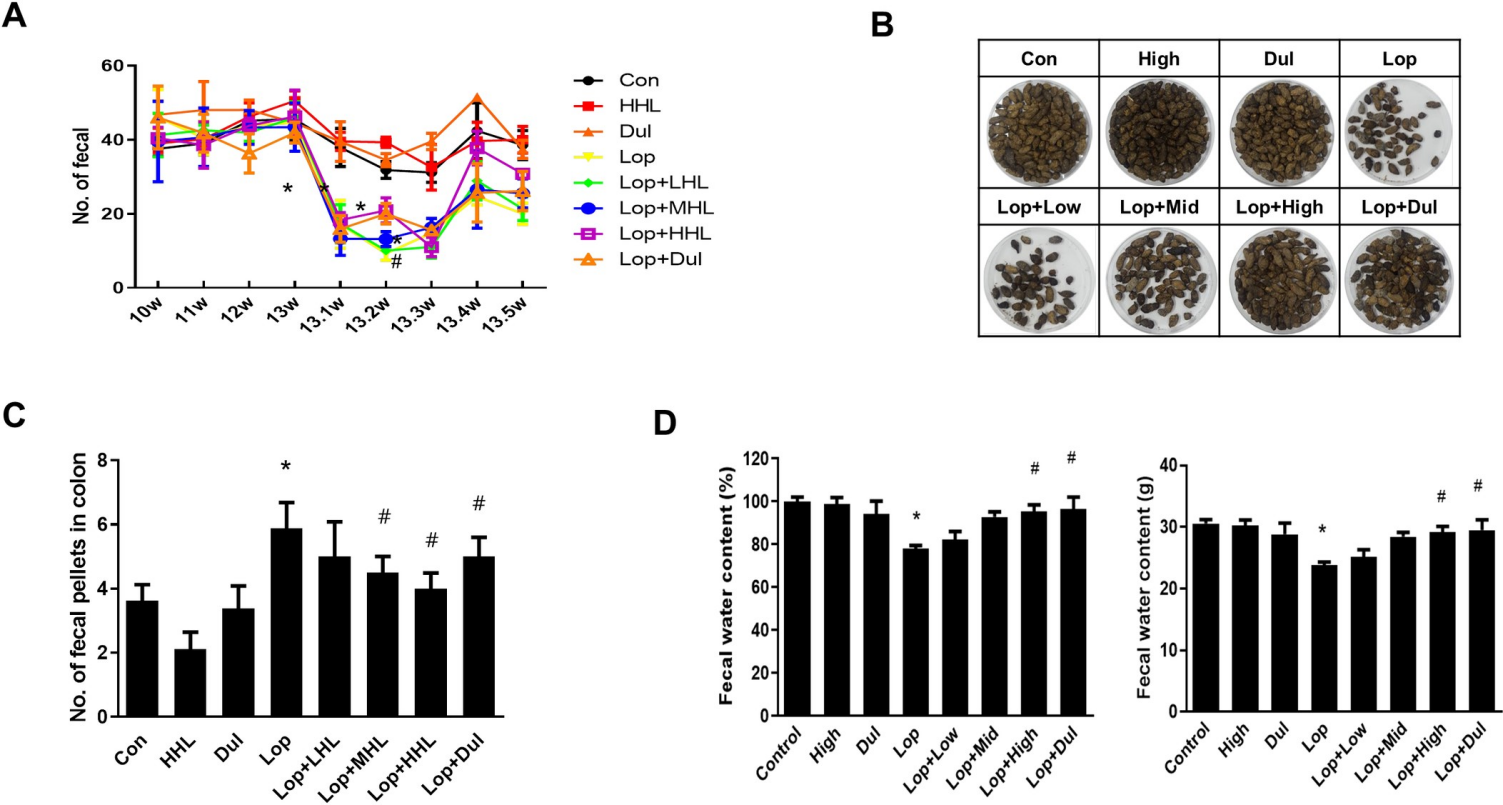
Effect of HLp-nF1 on constipation parameters. Eight-week-old male rats were treated with loperamide along with 3.2 × 10^10^, 8 × 10^10^, and 1.6 × 10^11^ cells/mL HLp-nF1, and Dulcolax individually. A single treatment with 1.6 × 10^11^ cells/mLHLp-nF1 or Dulcolax was used as the control group. (**A**) The fecal pellets were counted at the indicated time points. (**B**) The fecal pellets collected prior to sacrifice, (**C**) the number of remaining fecal pellets in the colon, and (**D**) fecal water content is shown. Each value represents the mean ± SD. A t-test was used to determine the means of two groups (*n = 11*). *P< 0.05 vs. control group, ^#^*P*< 0.05 vs. loperamide-treated group. *Con*, control group; *Lop*, loperamide-treated group; *Dul*, Dulcolax-treated group (0.75 mg/kg); *HHL*, treatment with 1.6 × 10^11^ cells/mL HLp-nF1; *Lop+LHL*, treatment with loperamide and 3.2 × 10^10^ cells/mL HLp-nF1; *Lop+MHL*, treatment with loperamide and 8 × 10^10^ cells/mL HLp-nF1; *Lop+HHL*, treatment with loperamide and 1.6 × 10^11^ cells/mL HLp-nF1; *Lop+Dul* treated group.

Food and water intake in the constipation-induced rats subjected to HLp-nF1 treatment are shown in S1 Table. Food and water intake significantly decreased in the constipation-induced group than the control group, but rat’s bodyweight remained similar (S1B). Higher fasting blood glucose levels were observed in the constipation-induced group than other groups, including the HLp-nF1-treated group (S1C). Further, the number of excreted pellets every week, a representative marker of constipation, was decreased in the constipation-induced group but significantly restored dose-dependently upon treatment with HLp-nF1. The total amount of fecal pellets excreted during the experiment was higher in the HLp-nF1-treated group than the constipation-induced group (Fig 1A and 1B). Similarly, the colons residual fecal pellets were significantly higher in the constipation-induced group than the control. In contrast, in the HLp-nF1-treated group, residual fecal pellets comparatively decreased in a dose-dependent manner (Fig 1C). Moreover, the fecal water content was significantly lower in the constipation-induced group than the control group, whereas in the HLp-nF1-treated groups, it was virtually recovered to the basal level (Fig 1D). These observations indicate that the treatment of HLp-nF1 can alleviate constipation.

### Effect of HLp-nF1 on intestinal transit in constipation-induced rat model

**Fig 2:**
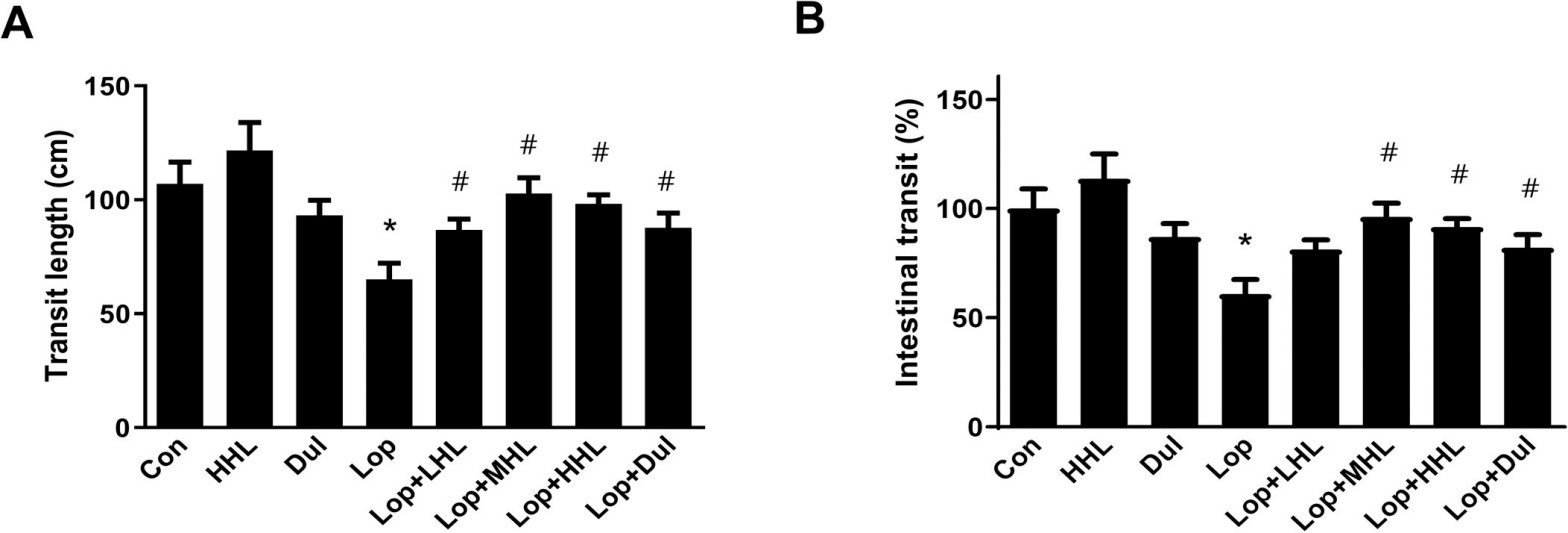
Effects of HLp-nF1 on a small intestinal transit parameter in a loperamide-induced constipation rat model. Eight-week-old male rats were treated with loperamide along with 3.2 × 10^10^, 8 × 10^10^, and 1.6 × 10^11^ cells/mLHLp-nF1, and Dulcolax, individually. A single treatment with 1.6 × 10^11^ cells/mLHLp-nF1 or Dulcolax was used as the control group. The intestinal transit length (**A**) and intestinal transit percentage (**B**) were measured and quantified as described in the Materials and Methods. The quantification analysis is shown. Data represent the mean ± SD. A t-test was used to determine the means of two groups (*n = 7*). *P< 0.05 vs. control group, ^#^*P*< 0.05 vs. loperamide-treated group. *Con*, control group; *Lop*, loperamide-treated group; *Dul*, Dulcolax-treated group (0.75 mg/kg); *HHL*, treatment with 1.6 × 10^11^ cells/mL HLp-nF1; *Lop+LHL*, treatment with loperamide and 3.2 × 10^10^ cells/mL HLp-nF1; *Lop+MHL*, treatment with loperamide and 8 × 10^10^ cells/mL HLp-nF1; *Lop+HHL*, treatment with loperamide and 1.6 × 10^11^ cells/mL HLp-nF1; *Lop+Dul* treated group.

Intestinal transit lengths, weight, and gastrointestinal motility are considered as intestine-associated function markers. In this study, both transit length and gastrointestinal motility were decreased in the constipation-induced groups, whereas both were recovered to the near-normal condition with the treatment of >3.2 × 10^10^ cells/mL HLp-nF1 (Fig 2A and 2B). However, the intestine length was not significantly changed with any treatment (S2A Fig). In the constipation-induced group, the intestinal weight was lower than the control and HLp-nF1, or Dul treated groups. However, there was no statistical significance (S2B Fig). These observations indicate that HLp-nF1 administration effectively reduces intestinal transit time, inhibits intestinal motility, and increases intestinal water uptake.

### Influence of HLp-nF1 on inflammation in the constipation model

**Fig 3:**
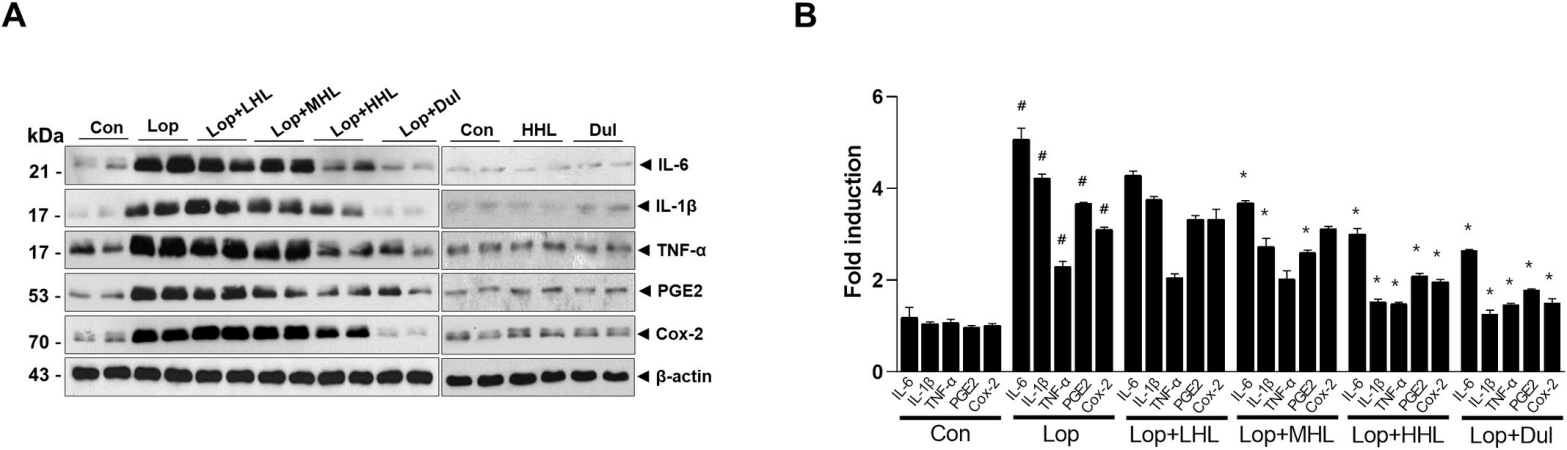
Effects of HLp-nF1 on inflammation state in loperamide-induced constipation. Eight-week-old rats were treated with loperamide along with 3.2 × 10^10^, 8 × 10^10^, and 1.6 × 10^11^ cells/mL HLp-nF1, and Dulcolax, individually. A single treatment with 1.6 × 10^11^ cells/mL HLp-nF1 or Dulcolax was used as the control. (**A**) Immunoblotting for anti-IL-6, IL-1β, TNF-α, PGE2, COX-2, and β-actin antibody was performed using the ileum samples. (**B**) The expression of these proteins were quantified. A t-test was used to determine the means of two groups *(n = 4)*. *P< 0.05 vs. control group, ^#^*P*< 0.05 vs. loperamide-treated group. *Con*, control group; *Lop*, loperamide-treated group; *Dul*, Dulcolax-treated group (0.75 mg/kg); *HHL*, treatment with 1.6 × 10^11^ cells/mL HLp-nF1; *Lop+LHL*, treatment with loperamide and 3.2 × 10^10^ cells/mL HLp-nF1; *Lop+MHL*, treatment with loperamide and 8 × 10^10^ cells/mL HLp-nF1; *Lop+HHL*, treatment with loperamide and 1.6 × 10^11^ cells/mL HLp-nF1; *Lop+Dul* treated group.

Serum cytokines levels were measured to evaluate the influence of HLp-nF1 on intestine inflammation. The levels of serum TNFα, IFNγ, IL-1β, IL-6, and PGE2 were increased in the constipation-induced group than in the control group (S2 Table). However, the level of serum IL-10 and IL-12 were decreased in the constipation-induced group compared to the control group and were recovered with the administration of HLp-nF1 or Dulcolax (Dul). There was no significant difference in the expression of serum IL-12 among the groups. The intestinal expression of IL-6, IL-1β, TNFα, PGE2, and COX-2 were increased in the constipation-induced group, whereas in the groups treated with HLp-nF1 or Dul, they were significantly decreased (Fig 3A and 3B). Additionally, lipid profile parameters were measured to evaluate the overall lipid metabolism in constipation (S2 Table). In the constipation-induced group, triglyceride and total cholesterol were increased, but the content of triglyceride was decreased in the group that was administered Lop with 8 × 10^10^ cells/mL HLp-nF1. Furthermore, the total cholesterol was also decreased by the Lop treatment and 3.2 × 10^10^ cells/mL HLp-nF1 than the constipation-induced group.

### Effect of HLp-nF1 on constipation-associated intestine histological alterations

**Fig 4:**
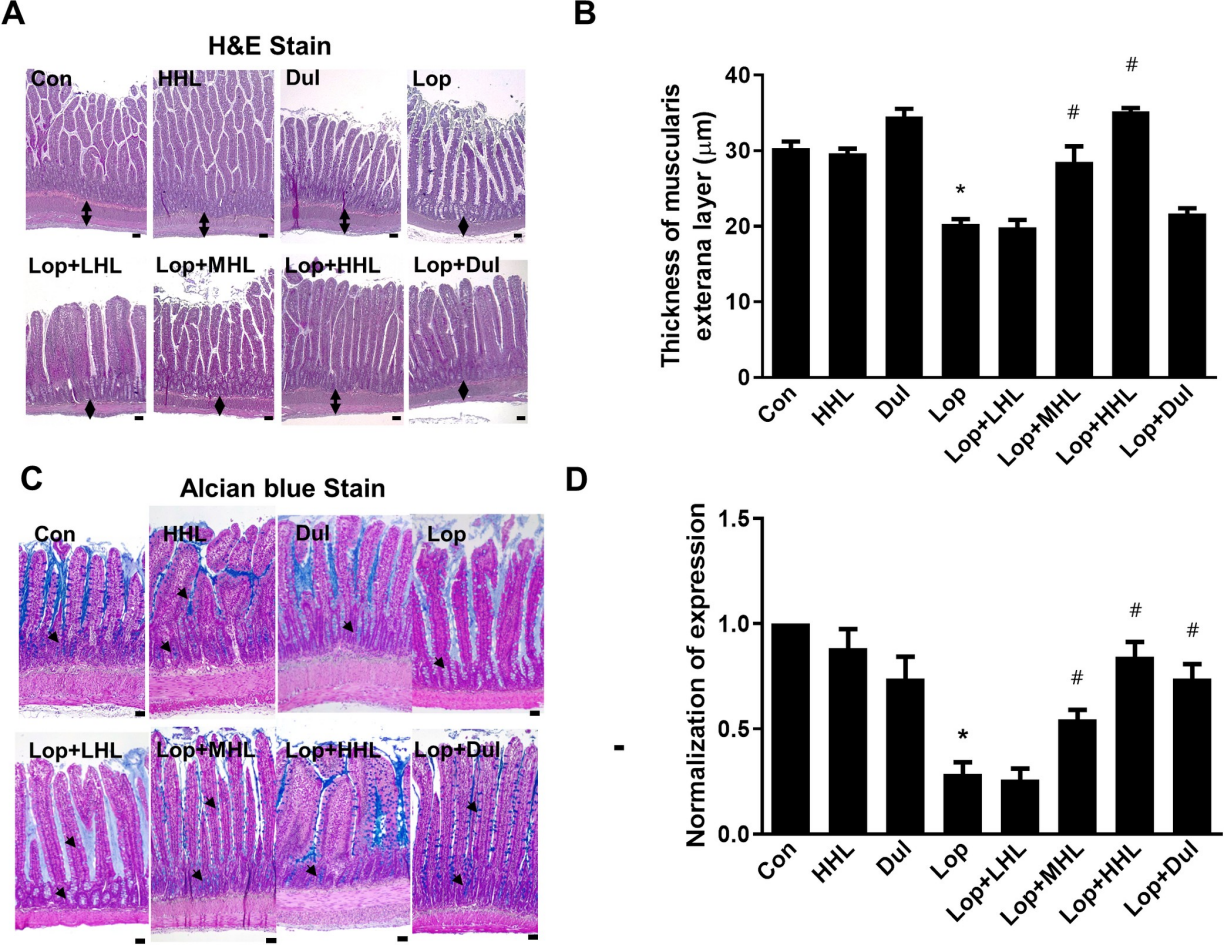
Effects of HLp-nF1 on the histological parameters in rats with loperamide-induced constipation. Eight-week-old rats were treated with loperamide along with 3.2 × 10^10^, 8 × 10^10^, and 1.6 × 10^11^ cells/mL HLp-nF1, and Dulcolax individually. A single treatment with 1.6 × 10^11^ cells/mL HLp-nF1 or Dulcolax was used as the control. (**A**) H&E-staining of the ilium and (**B**) the thickness of the muscularis externa layer was analyzed. (**C**) Alcian blue staining was performed as described in Materials and Methods, and (**D**) blue positive-mucin expression was quantified. A t-test was used to determine the means of two groups (*n = 3*). *P< 0.05 vs. control group, ^#^*P*< 0.05 vs. loperamide-treated group. Scale bar, 10 μm. *Con*, control group; *Lop*, loperamide-treated group; *Dul*, Dulcolax-treated group (0.75 mg/kg); *HHL*, treatment with 1.6 × 10^11^ cells/mL HLp-nF1; *Lop+LHL*, treatment with loperamide and 3.2 × 10^10^ cells/mL HLp-nF1; *Lop+MHL*, treatment with loperamide and 8 × 10^10^ cells/mL HLp-nF1; *Lop+HHL*, treatment with loperamide and 1.6 × 10^11^ cells/mL HLp-nF1; *Lop+Dul* treated group.

A decrease in mucous layer thickness and the resultant alterations in mucin synthesis and release are considered the main pathological mechanisms involved in constipation [15]. In this study, reduced thickness in the intestinal muscularis externa layer was observed in the constipation-induced group, and the administration of HLp-nF1 increased the thickness dose-dependently (Fig 4A and 4B). Additionally, mucin’s intensity in mucosal layer was decreased in the constipation-induced group compared with that in the control group. On the contrary, with the treatment of >3.2 × 10^10^ cells/mL HLp-nF1 and positive control groups (Dul), the mucus staining pattern was markedly observed, indicating the improvements (Fig 4C and 4D).

### Influence of HLp-nF1 on contraction of intestine

**Fig 5:**
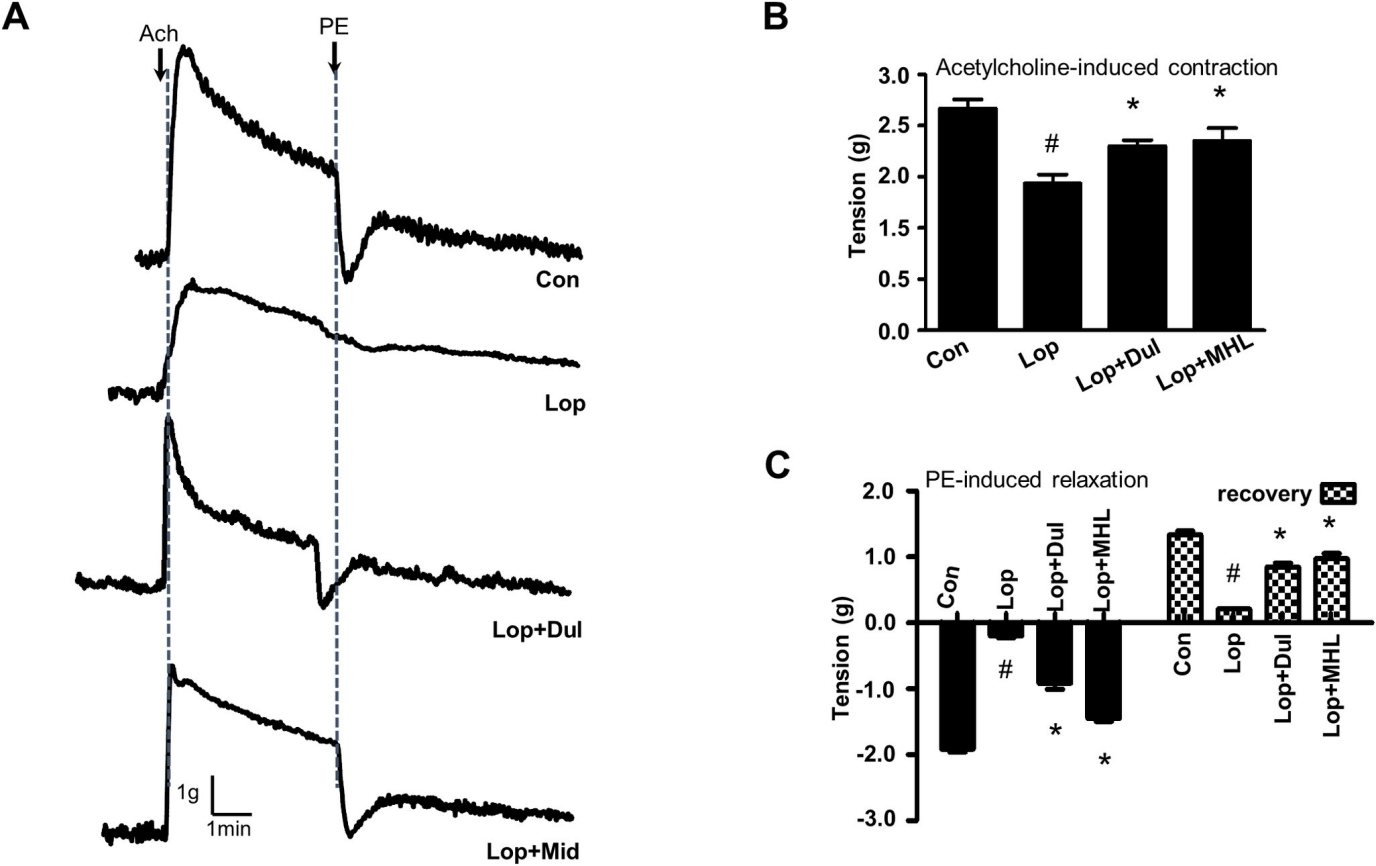
Effect of HLp-nF1 on intestinal contractility. Eight-week-old male rats were treated with loperamide with or without 8 × 10^10^ cells/mL HLp-nF1 and Dulcolax individually. (**A**) After isolation from the treated rats, the ileum was treated with 1 μM acetylcholine, and then with 1 μM phenylephrine. (**B**) Quantification of acetylcholine-induced intestine contraction. (**C**) Quantification of phenylephrine-induced intestine relaxation and recovery. A t-test was used to determine the means of two groups (*n = 4*). *P< 0.05 vs. control group, ^#^*P*< 0.05 vs. loperamide-treated group). *Con*, control group; *Lop*, loperamide-treated group; *Lop+MHL*, treatment with loperamide and 8 × 10^10^ cells/mL HLp-nF1; *Lop+Dul* treated group.

Contraction of intestine is a representative marker for efficacy against constipation. Therefore, acetylcholine (ACh) and phenyl epinephrine-induced contraction and relaxation were measured. A relatively similar acetylcholine-induced contraction was observed in control, Dul, and medium-dose HLp-nF1 (8 × 10^10^ cells/mL)-treated constipation-induced groups. The treatment with HLp-nF1 showed high contractibility, similar to that of Lop+Dul treatment (Fig 5A). In Ach presence, the ileum tension was reduced in the constipation-induced group, which was recovered by the treatment with Lop+Dul or Lop+MHL (Fig 5B). Phenylalanine-induced relaxation was impaired in the constipation-induced group, whereas the relaxation was significantly recovered in the Lop+MHL and Lop+Dul treated groups (Fig 5C).

### Alteration in microbiome upon administration of HLp-nF1

**Fig 6:**
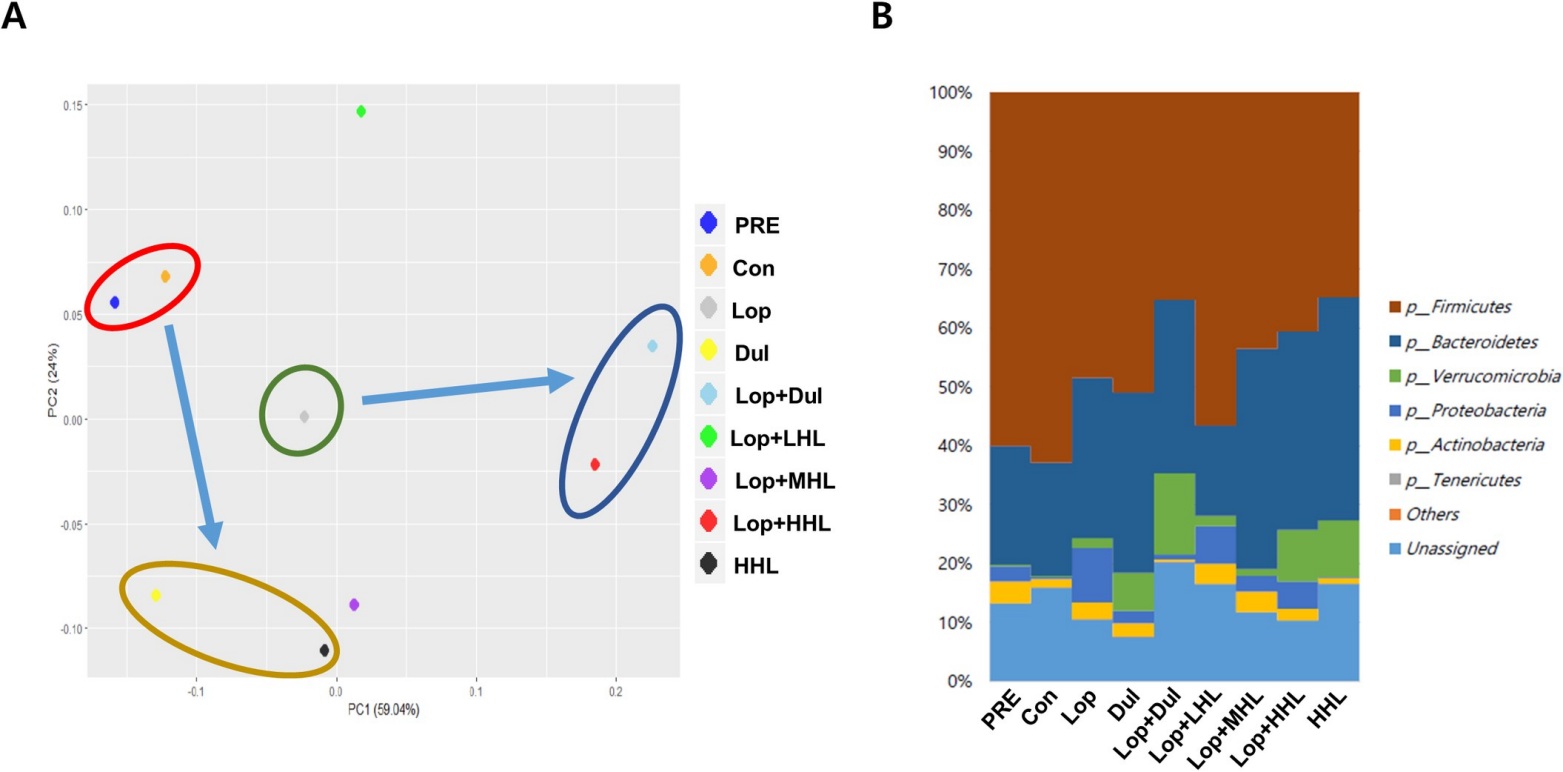
Changes of the genus pattern and microbiome composition in Extracellular Vesicles (EVs) and bacteria in rat feces. Eight-week-old rats were treated with loperamide along with 3.2 × 10^10^, 8 × 10^10^, and 1.6 × 10^11^ cells/mL HLp-nF1, and Dulcolax, individually. A single treatment with 1.6 × 10^11^ cells/mL HLp-nF1 or Dulcolax was used as the control. (**A**) The genus pattern in each group is graphically shown. The average relative density of the dominant bacterial phyla. (**B**) The X-axis shows the groups, and the Y-axis displays the average percentage of sequence reads. The cutoff point for selecting the dominant phyla was set at ≥ 1%. “Others” indicates minor phyla, and “Unassigned” indicates non-identified phyla. PRE, pre-preparation group; *Con*, control group; *Lop*, loperamide-treated group; *Dul*, Dulcolax-treated group (0.75 mg/kg); *HHL*, treatment with 1.6 × 10^11^ cells/mL HLp-nF1; *Lop+LHL*, treatment with loperamide and 3.2 × 10^10^ cells/mL HLp-nF1; *Lop+MHL*, treatment with loperamide and 8 × 10^10^ cells/mL HLp-nF1; *Lop+HHL*, treatment with loperamide and 1.6 × 10^11^ cells/mL HLp-nF1; *Lop+Dul* treated group.

MiSeq analysis was performed using the feces samples to determine the change in the distribution of intestinal bacteria and the microbiota type upon HLp-nF1 treatment. The principal component analysis (PCA) results of the genus analysis revealed that the constipation-induced group was distinctly different from both the pre-preparation (PRE) and control groups, showing a significant change in the genus pattern in the presence of a high dose of HLp-nF1 and Dul (Fig 6A). There was a similar genus pattern in both the PRE and control groups. Besides, similar patterns were observed among the Dul- and high-dose HLp-nF1-treated groups. Low dose showed a different pattern, not included in any other category. The results also showed that the lineage of Firmicutes, Proteobacteria, Actinobacteria, Bacteroidetes, and Verrucomicrobia dominated, as shown by their relatively high density in the feces (Fig 6B). Firmicutes and Bacteroidetes were dominant in all the groups, including the normal control group. In the HLp-nF1-treated groups, the density of Firmicutes was decreased in a dose-dependent manner. In contrast, the density of bacteroidetes was increased in the medium-dose and high-dose HLp-nF1-treated groups. Besides, Verrucomicrobia density was increased in the high-dose HLp-nF1-treated group, whereas Proteobacteria density was decreased in the medium-dose HLp-nF1-treated group of constipation. The percentage of the dominant phyla was also modulated by HLp-nF1 administration; however, the change was statistically not significant.

## Discussion

Probiotics are a highly appreciated option to restore and maintain balanced gut microbiota, and its usage is widespread across different regions of the world. However, several issues are reported against the use of live microorganisms, especially with vulnerable populations. Thus, the application of inactivated strains or dead cells have been investigated, and many of the studies are successful. The current study was aimed to examine the anti-constipation effect of HLp-nF1. Study observations have revealed that the administration of HLp-nF1 in constipated rats significantly improved the adverse effects of constipation, indicating the potential anti-constipation effect of HLp-nF1.

Probiotics containing nonpathogenic live microorganisms are used in a specified amount to provide health benefits to the host; however, safety concerns were on the rise in certain clinical conditions [6] due to translocation of gut microbiota to the systemic circulation. On the other side, several investigations suggest that heat-inactivated cells release crucial components that act like live cells and exert relevant biological responses [6, 9, 10]. These positive outcomes have triggered widespread investigation on inactivated cells or dead cells. Additionally, inactivated cells are safe as there are no translocation risks, acquisition of resistance, and interference with normal colonization. Thus, the beneficial effect and lower risk factors drove the current investigation. Essentially, the administration of HLp-nF1 significantly improved the excretion and fecal water content, which distinctly suggested the anti-constipation effect of HLp-nF1 (Fig 1A–1D). In line with these observations, previous studies on the heat-treated *Lactobacillus* revealed positive implications on the functions of the intestinal tract [13]. Also, few other investigations indicated the positive benefits of heat-killed bacteria in animal models and clinical trials [9, 21, 22].

Improvement in the excretion and fecal water content in the current investigation virtually suggests the anti-constipation effect of HLp-nF1. However, there is a need to understand the underlying modifications to confirm the anti-constipation effect of HLp-nF1. In vivo, the colonic measurement might be handy to support the anti-constipation effect of HLp-nF1, and several methods have been previously used to assess the transit times and motility. X-ray, scintigraphic techniques, ultrasound, and magnetic field detectors are the most preferred ones. However, all these methods have limitations such as low temporal resolution, partial information, and orally ingested capsules are foreign in nature, which does not reflect the GI tract’s precise physiology. Also, advanced techniques such as MRI are not cost-effective [23]. To overcome these constraints, inflammation parameters, including cytokine release, were evaluated in this study. Previously, *Lactobacillus sp* administered as probiotic was demonstrated to have an anti-inflammation effect [24]. During intestinal inflammation, pro-inflammatory cytokines such as IL-1β and TNFα induced a pronounced suppression of the release of acetylcholine and noradrenaline [25, 26], which can be linked to inflammation and contractility. During constipation, cytokines and the linked imbalanced nutrients are expected to negatively affect intestinal epithelial barrier integrity, amplifying the inflammation status [27–32]. In the present study, treatment with HLp-nF1 seems to have controlled constipation-associated pro-inflammatory cytokine release. It was observed that expression of serum TNFα, IFNγ, IL-1β, IL-6, and prostaglandin E2 were highly expressed in the constipated-induced group, whereas the same was less expressed with the administration of HLp-nF1 (Fig 3A and 3B).

Glucose and lipid profiles, triglyceride, and cholesterol levels increased in the constipation condition, whereas the profiles were significantly recovered in the probiotic-treated condition (S1A Fig and S2 Table). Though these parameters may not be involved in constipation directly, there could be potential indirect involvement. Interestingly, in a clinical study related to IBD, total cholesterol levels were lower than healthy individuals and higher in patients undergoing remission [33]. High glucose levels were observed in the constipated rat than other groups as inflammation is linked to the onset of diabetes. Specifically, few clinical observations have indicated that inflammatory bowel disease (IBD) can lead to the development of type 2 diabetes [34]. Hence, higher inflammation in constipated rats might have caused high blood glucose levels. Also, *Lactobacillus* administration has shown improved glycemic response, specifically in type 2 diabetes mellitus [35, 36]. Thus, glucose levels and lipid profiles potentially affect constipation, and observations in the study suggest that metabolic disturbances and the inflammation state can also be controlled with HLp-nF1.

Further, HLp-nF1 administered rats were observed to have a thickened intestinal mucosa layer, which itself indicates the beneficial effect (Fig 4A and 4B). A decrease in mucous layer thickness and the resultant alterations in mucin synthesis and release are considered pathological mechanisms involved in constipation [15]. Intestinal motility and intestinal function parameters were also evaluated to verify the anti-constipation effect of HLp-nF1. It is well known that contraction and relaxation are key processes for a smoother bowel movement [37, 38]. HLp-nF1 administration improved the ileum relaxation and its resilience in the present study, indicating the positive influence on contractility status associated with constipation-induced motility alteration (Fig 5A and 5C). These observations collectively signal lower inflammation levels in HLp-nF1 -treated groups and a possible positive impact on contractility. Consistent with these observations, some trials suggest that probiotics are the safe and better option for treating diarrhea [39]. Moreover, a complex interaction between inflammation and intestine barrier integrity is suggested to influence the gut microbiota. This interaction opens up the possibility of intestine microbiome environment altercations.

Human gastrointestinal (GI) tract harbors dynamic populations of microorganisms, and multiple factors influence the microbial composition. Interestingly, microbiome data revealed that the HLp-nF1 administration affected the population of intestine bacteria. Intestinal epithelium sense risk signals and luminal antigens through a group of specialized recognition receptors and pass the information to immune cells. Antibodies surround the mucus layer, and antimicrobial peptides and epithelium are physically separated from direct contact with luminal microbiota [40]. In the present study, pretreatment of rats and control groups showed similar patterns. In contrast, constipation-induced groups treated with Dul and a high dose of HLp-nF1 showed similar patterns in the comparative microbiome analysis (Fig 6). This observation indicates that treatment with HLp-nF1 does not alter the microbial compositions significantly. However, *Lactobacillus* relative ratio in the constipation-induced groups treated with Dul and a high dose of HLp-nF1 was lower than that in other experimental groups or control groups. Dysbiosis has been associated with the pathogenesis of several inflammatory diseases and infections. However, in this study, the microbial composition was not studied in-depth, but administration of HLp-nF1 revealed beneficial effect. Although it is difficult to explain the classification of beneficial and harmful bacteria uniformly, it is suggested that changes in the intestinal bacterial species can be attributed to the administration of HLp-nF1. These variations in species diversity might induce modifications in bacterial density as well as its gross weight. Change in density can contribute to change in whole intestine weight [41]. Similarly, reduced intestinal weight was observed in the constipation-induced group than the HLp-nF1 administration. In support of these observations, Warda et al. study revealed similar observations where heat-killed lactobacilli altered both microbiota and composition in mice model [42]. Also, these beneficial effects match the new concept of postbiotics [43]. Together, microbiota observations indicate that the administration of HLp-nF1 achieves changes in the microbiota and that intestinal health can be maintained through more stable ingestion and secretion. Investigative evidence reveals similar observations where they indicate that dysbiosis of gut microbiota potentially contributes to functional constipation and IBS [44]. Accordingly, most investigations have suggested that probiotics are one of the options to treat constipation. Apart from constipation, they also contribute to the enhanced immune system, reduce serum cholesterol, cancer prevention, antihypertensive effects and improve lactose metabolism [45].

In conclusion, the results demonstrate that Lop-induced constipation was alleviated by administering HLp-nF1 to rats. Groups that have received >3.2 × 10^10^ cells/mL HLp-nF1 ameliorated the fecal parameters such as pellet number, weight, and water content. Also bettered the thickness of the mucin area in the intestine. HLp-nF1 improved contraction and relaxation of the ileum and the inflammation state such as inflammatory cytokines were also regulated in the >3.2 × 10^10^ cells/mL HLp-nF1 -treated group. The results strongly suggest that HLp-nF1 has the potential to be developed as a therapeutic and preventive strategy for constipation.

## Supporting information

S1 Fig Experimental scheme, body weight and glucose levels during study period. **(A)** Schematic representation of the experimental scheme. Effect of HLp-nF1 on the body weight and fasting glucose level. Eight-week-old rats were treated with loperamide, and then with 3.2 × 10^10^, 8 × 10^10^, and 1.6 × 10^11^ cells/mL HLp-nF1, and Dulcolax, individually. A single treatment with 1.6 × 10^11^ cells/mL HLp-nF1 or Dulcolax was used as the control. The body weight (**B**) and serum glucose level (**C**) were measured. Each value is mean ± SD. *P< 0.05 vs. *Con*, control group; *Lop*, loperamide-treated group; *Dul*, Dulcolax-treated group; *HHL*, treatment with 1.6 × 10^11^ cells/mL HLp-nF1; *Lop+LHL*, treatment with loperamide and 3.2 × 10^10^ cells/mL HLp-nF1; *Lop+MHL*, treatment with loperamide and 8 × 10^10^ cells/mL HLp-nF1; *Lop+HHL*, treatment with loperamide and 1.6 × 10^11^ cells/mL HLp-nF1; *Lop+Dul* treated group.
(DOCX)

S2 Fig Effect of HLp-nF1 on the intestine length and weight in loperamide-induced constipation. Eight-week-old rats were treated with loperamide, and then with 3.2 × 10^10^, 8 × 10^10^, and 1.6 × 10^11^ cells/mL HLp-nF1, and Dulcolax, individually. A single treatment with 1.6 × 10^11^ cells/mL HLp-nF1 or Dulcolax was used as the control. The intestine length (**A**, measurement in the right) and weight (**B**) were measured. *Con*, control group; *Lop*, loperamide-treated group; *Dul*, Dulcolax-treated group; *HHL*, treatment with 1.6 × 10^11^ cells/mL HLp-nF1; *Lop+LHL*, treatment with loperamide and 3.2 × 10^10^ cells/mL HLp-nF1; *Lop+MHL*, treatment with loperamide and 8 × 10^10^ cells/mL HLp-nF1; *Lop+HHL*, treatment with loperamide and 1.6 × 10^11^ cells/mL HLp-nF1; *Lop+Dul* treated group.
(DOCX)

S3 Fig Effect of dextrin and HLp-nF1 on loperamide-induced constipation. The isolated ileum from eight-week-old rats was treated with cluster dextrin, 1 μM acetylcholine (Ach), and subsequently 1 μM phenylephrine (PE). There was no change in the response curve trace with the accumulative the addition of cluster dextrin to the ileum. (**A**) Representative tracings showed that 1 μM Ach-induced contraction of rat ileum was after the dextrins accumulative addition. Non-response of cluster dextrin in the ileum. (**B**) Representative tracings showed that the relaxation of rat ileum was induced by the cumulative addition of cluster dextrin after 1-μM-Ach-induced contraction, followed by the application of 1 μM PE. *1*, 0.23 mg/mL; *2*, 0.46 mg/mL; *3*, 0.92 mg/mL; *4*, 4.6 mg/mL; *5*, 9.2 mg/mL; and *6*, 18.4 mg/mL.
(DOCX)

S4 Fig Alteration in microbiome upon administration of HLp-nF1. (A) Phyla observations obtained through MiSeq analysis. (B) Genus observations obtained through MiSeq analysis. PRE, pre-preparation group; *Con*, control group; *Lop*, loperamide-treated group; *Dul*, Dulcolax-treated group (0.75 mg/kg); *HHL*, treatment with 1.6 × 10^11^ cells/mL HLp-nF1; *Lop+LHL*, treatment with loperamide and 3.2 × 10^10^ cells/mL HLp-nF1; *Lop+MHL*, treatment with loperamide and 8 × 10^10^ cells/mL HLp-nF1; *Lop+HHL*, treatment with loperamide and 1.6 × 10^11^ cells/mL HLp-nF1; *Lop+Dul* treated group.
(DOCX)

S1 Raw images Uncropped and unadjusted images underlying blot results revealing effects of HLp-nF1 on inflammation state in loperamide-induced constipation. (DOCX)

S1 Table Effects of HLp-nF1 on food and water intake in loperamide-induced constipated rats. (DOCX)

S2 Table Effects of HLp-nF1 on inflammation signal in loperamide-induced constipated rats. (DOCX)
