# Supplementary material for: Heat-inactivated Lactobacillus plantarum nF1 promotes intestinal health in Loperamide-induced constipation rats
Source: PLoS One. 2021 Apr 19;16(4):e0250354. doi: 10.1371/journal.pone.0250354 (PMC8055018; doi:10.1371/journal.pone.0250354)
Supplement: S1 Fig — (A) Schematic representation of the experimental scheme. Effect of HLp-nF1 on the body weight and fasting glucose level. Eight-week-old rats were treated with loperamide, and then with 3.2 × 10^10^, 8 × 10^10^, and 1.6 × 10^11^ cells/mL HLp-nF1, and Dulcolax, individually. A single treatment with 1.6 × 10^11^ cells/mL HLp-nF1 or Dulcolax was used as the control. The body weight (B) and serum glucose level (C) were measured. Each value is mean ± SD. *P< 0.05 vs. Con, control group; Lop, loperamide-treated group; Dul, Dulcolax-treated group; HHL, treatment with 1.6 × 10^11^ cells/mL HLp-nF1; Lop+LHL, treatment with loperamide and 3.2 × 10^10^ cells/mL HLp-nF1; Lop+MHL, treatment with loperamide and 8 × 10^10^ cells/mL HLp-nF1; Lop+HHL, treatment with loperamide and 1.6 × 10^11^ cells/mL HLp-nF1; Lop+Dul treated group. (DOCX) [file pone.0250354.s001.docx]

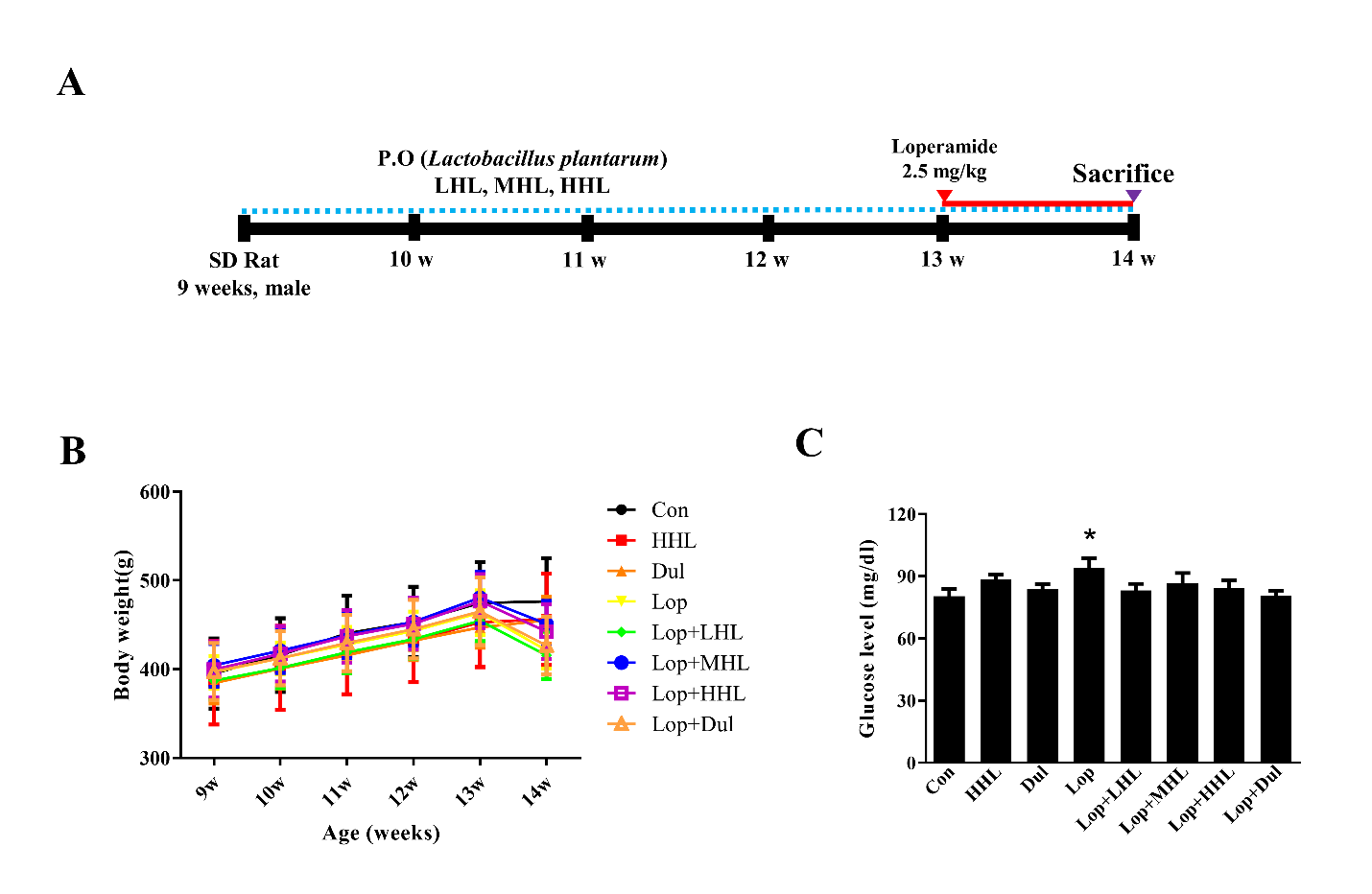


**S1 Fig.** **(A)** Schematic representation of the experimental scheme*.* Effect of HLp-nF1 on the body weight and fasting glucose level. Eight-week-old rats were treated with loperamide, and then with 3.2 × 10^10^, 8 × 10^10^, and 1.6 × 10^11^ cells/mL HLp-nF1, and Dulcolax, individually. A single treatment with 1.6 × 10^11^ cells/mL HLp-nF1 or Dulcolax was used as the control. The body weight (**B**) and serum glucose level (**C**) were measured. Each value is mean ± SD. **P* < 0.05 vs. *Con,* control group; *Lop,* loperamide-treated group; *Dul,* Dulcolax-treated group; *HHL,* treatment with 1.6 × 10^11^ cells/mL HLp-nF1; *Lop+LHL,* treatment with loperamide and 3.2 × 10^10^ cells/mL HLp-nF1; *Lop+MHL,* treatment with loperamide and 8 × 10^10^ cells/mL HLp-nF1; *Lop+HHL,* treatment with loperamide and 1.6 × 10^11^ cells/mL HLp-nF1; *Lop+Dul* treated group.
