# Supplementary material for: Heat-inactivated Lactobacillus plantarum nF1 promotes intestinal health in Loperamide-induced constipation rats
Source: PLoS One. 2021 Apr 19;16(4):e0250354. doi: 10.1371/journal.pone.0250354 (PMC8055018; doi:10.1371/journal.pone.0250354)
Supplement: S3 Fig — The isolated ileum from eight-week-old rats was treated with cluster dextrin, 1 μM acetylcholine (Ach), and subsequently 1 μM phenylephrine (PE). There was no change in the response curve trace with the accumulative the addition of cluster dextrin to the ileum. (A) Representative tracings showed that 1 μM Ach-induced contraction of rat ileum was after the dextrins accumulative addition. Non-response of cluster dextrin in the ileum. (B) Representative tracings showed that the relaxation of rat ileum was induced by the cumulative addition of cluster dextrin after 1-μM-Ach-induced contraction, followed by the application of 1 μM PE. 1, 0.23 mg/mL; 2, 0.46 mg/mL; 3, 0.92 mg/mL; 4, 4.6 mg/mL; 5, 9.2 mg/mL; and 6, 18.4 mg/mL. (DOCX) [file pone.0250354.s003.docx]

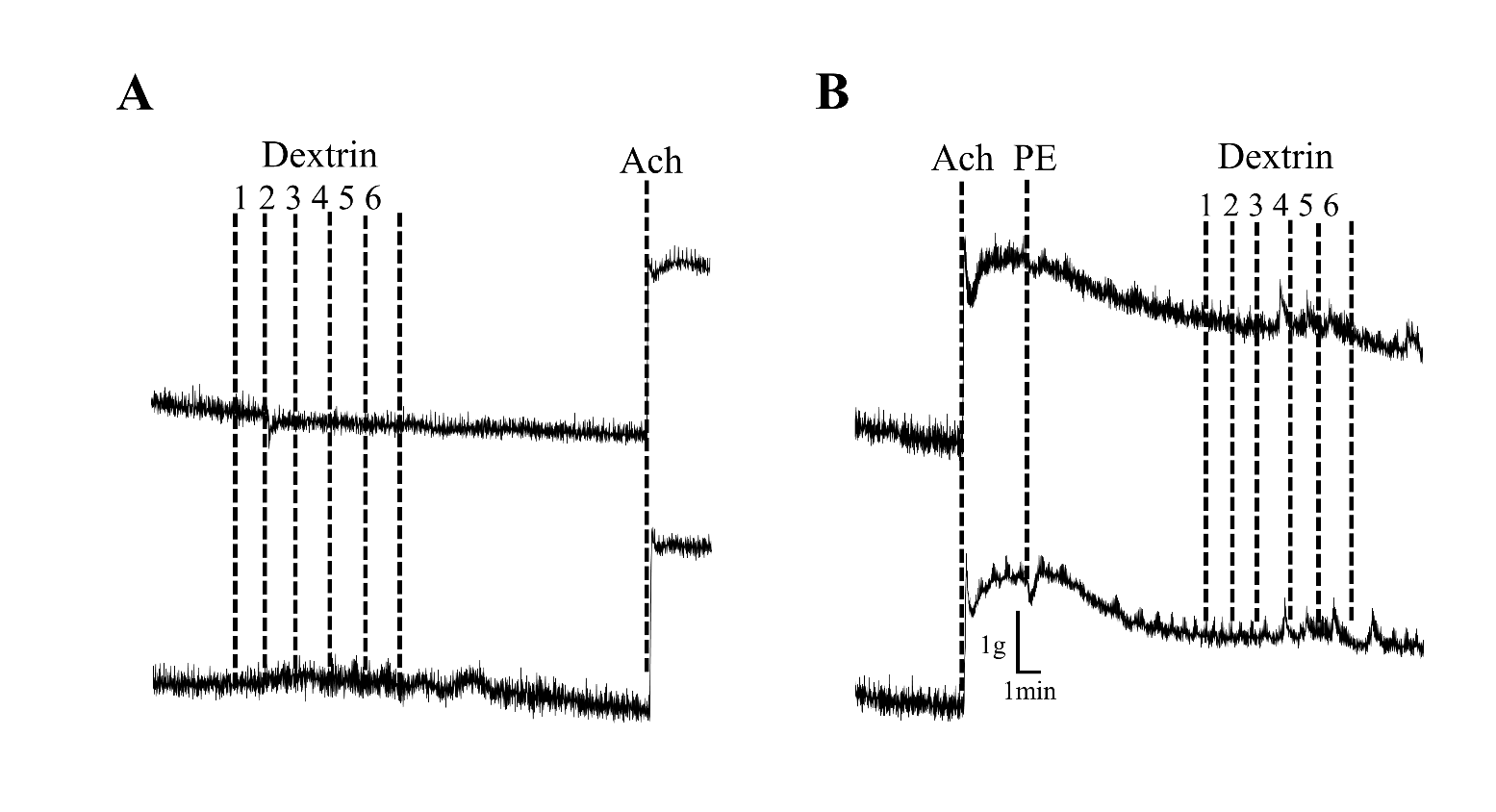


**S3 Fig.** Effect of dextrin and HLp-nF1 on loperamide-induced constipation. The isolated ileum from eight-week-old rats was treated with cluster dextrin, 1 μM acetylcholine (Ach), and subsequently 1 μM phenylephrine (PE). here was no change in the response curve trace with the accumulative the addition of cluster dextrin to the ileum. (**A**) Representative tracings showed that 1 μM Ach-induced contraction of rat ileum was after the dextrins accumulative addition. Non-response of cluster dextrin in the ileum. (**B**) Representative tracings showed that the relaxation of rat ileum was induced by the cumulative addition of cluster dextrin after 1-μM-Ach-induced contraction, followed by the application of 1 μM PE. *1*, 0.23 mg/mL; *2*, 0.46 mg/mL; *3*, 0.92 mg/mL; *4*, 4.6 mg/mL; *5*, 9.2 mg/mL; and *6*, 18.4 mg/mL.
