# Supplementary material for: Heat-inactivated Lactobacillus plantarum nF1 promotes intestinal health in Loperamide-induced constipation rats
Source: PLoS One. 2021 Apr 19;16(4):e0250354. doi: 10.1371/journal.pone.0250354 (PMC8055018; doi:10.1371/journal.pone.0250354)
Supplement: S4 Fig — (A) Phyla observations obtained through MiSeq analysis. (B) Genus observations obtained through MiSeq analysis. PRE, pre-preparation group; Con, control group; Lop, loperamide-treated group; Dul, Dulcolax-treated group (0.75 mg/kg); HHL, treatment with 1.6 × 10^11^ cells/mL HLp-nF1; Lop+LHL, treatment with loperamide and 3.2 × 10^10^ cells/mL HLp-nF1; Lop+MHL, treatment with loperamide and 8 × 10^10^ cells/mL HLp-nF1; Lop+HHL, treatment with loperamide and 1.6 × 10^11^ cells/mL HLp-nF1; Lop+Dul treated group. (DOCX) [file pone.0250354.s004.docx]

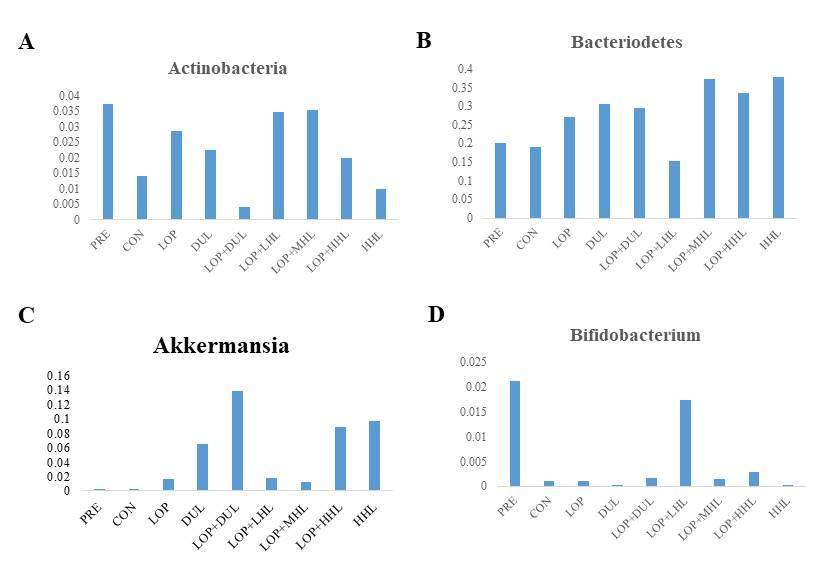


**S4 Fig.** **Alteration in microbiome upon administration of HLp-nF1.** . (A) Phyla observations obtained through MiSeq analysis. (B) Genus observations obtained through MiSeq analysis. PRE, pre-preparation group; *Con,* control group; *Lop,* loperamide-treated group; *Dul,* Dulcolax-treated group (0.75 mg/kg); *HHL,* treatment with 1.6 × 10^11^ cells/mL HLp-nF1; *Lop+LHL,* treatment with loperamide and 3.2 × 10^10^ cells/mL HLp-nF1; *Lop+MHL,* treatment with loperamide and 8 × 10^10^ cells/mL HLp-nF1; *Lop+HHL,* treatment with loperamide and 1.6 × 10^11^ cells/mL HLp-nF1; *Lop+Dul* treated group.
