# Supplementary material for: Heat-inactivated Lactobacillus plantarum nF1 promotes intestinal health in Loperamide-induced constipation rats
Source: PLoS One. 2021 Apr 19;16(4):e0250354. doi: 10.1371/journal.pone.0250354 (PMC8055018; doi:10.1371/journal.pone.0250354)
Supplement: S1 Raw images — (DOCX) [file pone.0250354.s005.docx]

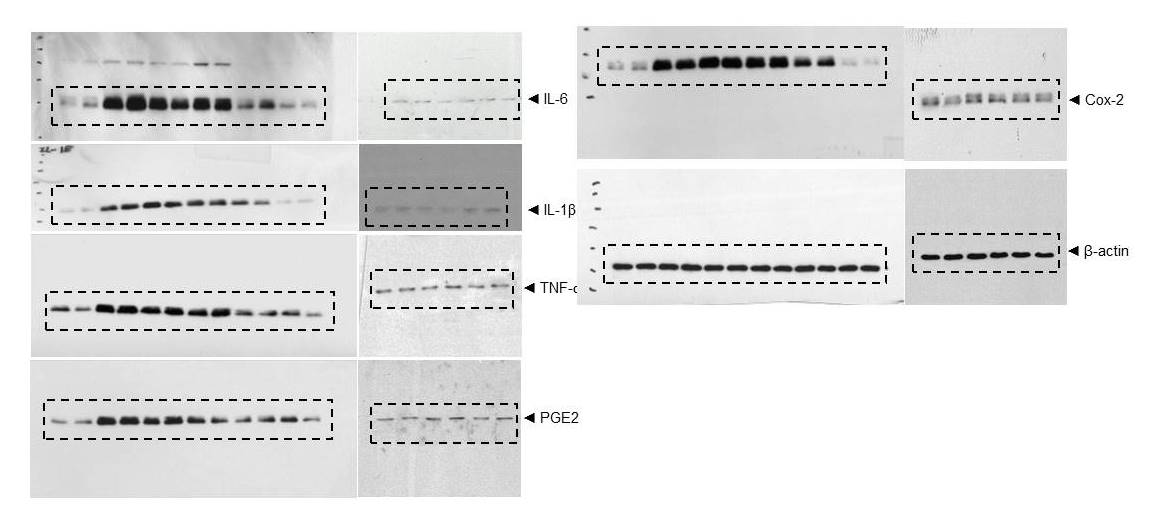


**S1 Raw images. Uncropped and unadjusted images underlying blot results revealing effects of HLp-nF1 on inflammation state in loperamide-induced constipation.**
