# Supplementary material for: Heat-inactivated Lactobacillus plantarum nF1 promotes intestinal health in Loperamide-induced constipation rats
Source: PLoS One. 2021 Apr 19;16(4):e0250354. doi: 10.1371/journal.pone.0250354 (PMC8055018; doi:10.1371/journal.pone.0250354)
Supplement: S1 Table — (DOCX) [file pone.0250354.s006.docx]

**S1 Table. Effects of HLp-nF1 on food and water intake in loperamide-induced constipated rats.**

|  | **Food intake (g/day)** | | **Water intake (mL/day)** | |
| --- | --- | --- | --- | --- |
|  | **Before constipation** | **After**  **constipation** | **Before**  **constipation** | **After**  **constipation** |
| **Con^1^** | **37 ± 1.9** | **37 ± 1.9** | **37 ± 2.9** | **40 ± 3.4** |
| **High^2^** | **35 ± 5.8** | **36 ± 3.1** | **36 ± 2.4** | **39 ± 2.6^*^** |
| **Dul^3^** | **37 ± 2.7** | **36 ± 2.9** | **35 ± 1.4** | **38 ± 2.0^*^** |
| **Lop^4^** | **37 ± 0.8** | **27 ± 2.4^*#^** | **41 ± 5.9** | **29 ± 0.4^*#^** |
| **Lop+LH^5^** | **35 ± 1.6** | **28 ± 1.1^*^** | **37 ± 0.6** | **30 ± 1.6^*##^** |
| **Lop+MH^6^** | **39 ± 0.8** | **30 ± 3.2** | **40 ± 3** | **34 ± 2.5^##^** |
| **Lop+HH** | **38± 3.7** | **32 ± 0.7^##^** | **36 ± 3** | **35 ± 0.9^##^** |
| **Lop+Dul^8^** | **37 ± 3.2** | **32 ± 1.8** | **37 ± 1.7** | **35 ± 1.3^##^** |

**P* < 0.05 vs. control group, ^#^*P* < 0.05 vs. loperamide-treated group, ^##^*P* < 0.05 vs before constipation. ^1^Con, control group; ^2^High, treatment with 1.6 × 10^11^ cells/mL HLp-nF1;^3^Dul, dulcolax-treated group; ^4^Lop, loperamide-treated group; ^5^Lop+LH, treatment with loperamide and 3.2 × 10^10^ cells/mL HLp-nF1; ^6^Lop+MH, treatment with loperamide and 8 × 10^10^ cells/mL HLp-nF1; ^7^Lop+HH, treatment with loperamide and 1.6 × 10^11^ cells/mL HLp-nF1; ^8^Lop+Dul, loperamide and Dulcolax treated group. Digits are rounded off to nearest digit.
