# Supplementary material for: Heat-inactivated Lactobacillus plantarum nF1 promotes intestinal health in Loperamide-induced constipation rats
Source: PLoS One. 2021 Apr 19;16(4):e0250354. doi: 10.1371/journal.pone.0250354 (PMC8055018; doi:10.1371/journal.pone.0250354)
Supplement: S2 Table — (DOCX) [file pone.0250354.s007.docx]

**S2 Table.** Effects of HLp-nF1 on inflammation signal in loperamide-induced constipated rats.

|  | | | | | **Group** | | | | | |
| --- | --- | --- | --- | --- | --- | --- | --- | --- | --- | --- |
|  | **Con^1^** | **High^2^** | **Dul^3^** | **Lop^4^** | | **Lop+LH^5^** | **Lop+MH^6^** | **Lop+HH^7^** | **Lop+Dul^8^** |  |
| **TNFα (pg/ml)** | **18 ± 3.5** | **19.7 ± 4** | **16.5 ± 2.4** | **21.6 ± 7.3^*^** | | **16 ± 2.2^*^** | **17 ± 2.5** | **18.3 ± 4.7** | **17 ± 2.9** |  |
| **IFNγ (pg/ml)** | **2.7 ± 0.46** | **3 ± 0.72** | **2.7 ± 0.53** | **3.7 ± 0.85^*^** | | **3.2 ± 0.72** | **3.2 ± 1.2^*^** | **3.5 ± 0.26** | **3.5 ± 1.3** |  |
| **IL1β (pg/ml)** | **14.8 ± 1.9** | **14 ± 3.3** | **12.5 ± 2.2** | **15.7 ± 3.7^*^** | | **13.5 ± 2.3** | **12.3 ± 3.1^*^** | **13.6 ± 2.4** | **14 ± 2.6** |  |
| **IL6 (pg/ml)** | **32.3 ± 1.5** | **32.6 ± 5** | **36.3 ± 2.9** | **52 ± 8.9^*^** | | **40± 6.7^*^** | **42.3 ± 7.3** | **44.3 ± 5** | **36.5 ± 7** |  |
| **IL12 (pg/ml)** | **421 ± 77** | **407 ± 101** | **506 ± 195** | **256± 107^*^** | | **262 ± 51** | **293 ± 43** | **315 ± 43^*^** | **214 ± 64** |  |
| **IL10 (pg/ml)** | **37.1 ± 5.6** | **39.5 ± 4** | **44 ± 11.51** | **21 ± 11.8^*^** | | **22.1 ± 9** | **32 ± 16.7** | **35 ± 15.4^*^** | **74 ± 22.3** |  |
| **Prostaglandin E2 (pg/ml)** | **5.25 ± 3.9** | **4.5 ± 5.1** | **12 ± 4.57** | **17.2 ± 6.5** | | **12.7 ± 4.6** | **8.8 ± 3.2** | **9.2 ± 3.7** | **17 ± 7.24** |  |
| **TG (mg/dl)** | **84 ± 10.3** | **80 ± 14.5** | **67.5 ± 8.3** | **89 ± 20^*^** | | **73 ± 8.33** | **69 ± 18.3^#^** | **72 ± 23.6** | **68.2 ± 8.9** |  |
| **Total**  **cholesterol (mg/dl)** | **183 ± 24** | **177 ± 9.9** | **170 ± 15** | **223 ± 20^*^** | | **194 ± 14^*^** | **194 ± 25** | **214 ± 14** | **201 ± 29** |  |

**P* < 0.05 vs. control group, ^#^*P* < 0.05 vs. loperamide-treated group. ^1^Con, control group; ^2^High, treatment with 1.6 × 10^11^ cells/mL HLp-nF1;^3^Dul, dulcolax-treated group; ^4^Lop, loperamide-treated group; ^5^Lop+LH, treatment with loperamide and 3.2 × 10^10^ cells/mL HLp-nF1; ^6^Lop+MH, treatment with loperamide and 8 × 10^10^ cells/mL HLp-nF1; ^7^Lop+HH, treatment with loperamide and 1.6 × 10^11^ cells/mL HLp-nF1. ^8^Lop+Dul, loperamide and Dulcolax treated group. Digits are rounded off to nearest digit.
